# Insect Behavior and Physiological Adaptation Mechanisms Under Starvation Stress

**DOI:** 10.3389/fphys.2019.00163

**Published:** 2019-03-05

**Authors:** Dao-Wei Zhang, Zhong-Jiu Xiao, Bo-Ping Zeng, Kun Li, Yan-Long Tang

**Affiliations:** School of Biological and Agricultural Science and Technology, Zunyi Normal University, Zunyi, China

**Keywords:** insect, starvation stress, behavior, trehalose, physiological adaptation, ecological regulation

## Abstract

Intermittent food shortages are commonly encountered in the wild. During winter or starvation stress, mammals often choose to hibernate while insects—in the form of eggs, mature larvae, pupae, or adults opt to enter diapause. In response to food shortages, insects may try to find sufficient food to maintain normal growth and metabolism through distribution of populations or even migration. In the face of hunger or starvation, insect responses can include changes in behavior and/or maintenance of a low metabolic rate through physiological adaptations or regulation. For instance, in order to maintain homeostasis of the blood sugar, trehalose under starvation stress, other sugars can be transformed to sustain basic energy metabolism. Furthermore, as the severity of starvation increases, lipids (especially triglycerides) are broken down to improve hunger resistance. Starvation stress simultaneously initiates a series of neural signals and hormone regulation processes in insects. These processes involve neurons or neuropeptides, immunity-related genes, levels of autophagy, heat shock proteins and juvenile hormone levels which maintain lower levels of physiological metabolic activity. This work focuses on hunger stress in insects and reviews its effects on behavior, energy reserve utilization, and physiological regulation. In summary, we highlight the diversity in adaptive strategies of insects to hunger stress and provides potential ideas to improve hunger resistance and cold storage development of natural enemy insects. This gist of literature on insects also broadens our understanding of the factors that dictate phenotypic plasticity in adjusting development and life histories around nutritionally optimal environmental conditions.

## Introduction

Food is a critical source of nutrients and an important external factor in insect survival. Lack of food over long periods of time affects the growth and reproduction of insects and may even result in death (Chang, 2015; Yang et al., 2016). However, insects can enter an anti-stress state to adapt to adverse conditions. Diapause, a decrease in metabolism, and increased lipid deposition help insects to adapt to food shortages and maintain homeostasis and recover once favorable conditions return (Sánchez-Paz et al., 2006; Buckemüller et al., 2017; McCue et al., 2017). Insects can enter diapause in the form of eggs, mature larvae, pupae or adults to survive the long and resource-scarce winter (Zhang X. Y. et al., 2015). In addition to natural seasonal changes, damage to the ecological environment can often lead to food shortages and is one of the most common and severe stresses in nature (Panizzi and Hirose, 1995; Rotkopf et al., 2013). Previous studies have found that insect responses to food deficiencies or hunger stress generally fall into one of the two categories: (1) behavioral activities, i.e., ecological adaptation strategies whereby insects find food-rich places through behaviors such as migration or other activities such as entry into diapause, etc. or (2) physiological countermeasures that regulate the metabolism of related physiological and biochemical substances in the body to improve the insect’s ability to endure hunger (Yang et al., 2016). Since food availability is crucial for insect survival, studying the consequences associated with starvation stress helps reveal adaptive strategies. Understanding these strategies is useful for the storage and release of natural insect enemies for biological pest control (Fujikawa et al., 2009). Previous reviews have summarized insect tolerance to starvation and hunger regulation mechanisms such as physiological adaptations to sugar intake (Chng et al., 2017), neurohormone adaptation (Perić-Mataruga, 2006) and diapause (Hahn and Denlinger, 2007). Here we review studies of insect behavioral and physiological adaption mechanisms under starvation, especially those involving energy changes between carbohydrates, lipids, and proteins, as well as nerve signals and hormone regulation.

## Regulation of Insect Behavior Under Starvation

Under the stress of starvation, insects tend to change their behavior that includes migration, cannibalism, early entry into diapause or the pupal stage, and reduced numbers of eggs (Table 1). For example, when larvae of *Drosophila melanogaster* (Diptera: Drosophilidae) and *Harmonia axyridis* (Coleoptera: Coccinellidae) are nutritionally challenged, they exhibit cannibalistic behavior (Ahmad et al., 2015). The main behavioral responses toward seasonal food shortages are migration and diapause. The rate at which larvae secrete ecdysteroids is significantly increased because hunger affects the level and production of ecdysone (Chen and Gu, 2006). Thus, larvae enter the pupal stage earlier under starvation conditions and the duration of pupation is prolonged with increasing days of starvation (Ballard et al., 2008). Moreover, pupae are very resistant so many insects endure adverse environmental conditions in the pupal form (Zhang X. Y. et al., 2015). For example, the larvae of *Psacothea hilaris* (Coleoptera: Cerambycidae) reach the pupal stage early after fasting (Munyiri and Ishikawa, 2005; Helm et al., 2017). When food is scarce, some beetles begin metamorphosis earlier (Zauner et al., 2000) while *Bactrocera dorsalis* (Diptera: Tephritidae) larvae enter the pupal stage after 12 h of starvation (Cong et al., 2015).

**Table 1:** Some insect behaviors and physiological adaptions under starvation conditions.

No.	Species name	Main behavioral regulation	Molecular and biochemical regulation	Hunger emergency response	Reference
1	*Drosophila melanogaster*	Cannibalism	Trehalose, glycogen	Physiological regulation	Parkash et al., 2012; Ahmad et al., 2015
2	*Harmonia axyridis*	Diapause	Trehalose, glycogen, proteins	Physiological regulation	Tang et al., 2014; Shen et al., 2015; Shi et al., 2017
3	*Psacothea hilaris*	Enter the pupal stage earlier	Trehalose, glucose	Physiological regulation	Munyiri and Ishikawa, 2005
4	*Bactrocera dorsalis*	Enter the pupal stage earlier	Octopamine	Physiological regulation	Yang, 2014; Cong et al., 2015; Chen et al., 2017; Hou et al., 2017; Li, 2017
5	*Apis mellifera* L.	Reduced reproductive capacity	Glycogen	Physiological regulation	Wang et al., 2016a,b
6	*Arma chinensis*	Fecundity and egg hatchability decrease	-	-	Zhang et al., 2017
7	*Eriosoma lanigerum*	Spawn in the early stages, migration	-	Escape	Chen, 2013
8	*Mythimna separate*	Migration	-	Escape	Li, 1993
9	*Trogoderma granarium*	-	Trehalose, glycogen	Physiological regulation	Mohammadzadeh and Izadi, 2018
11	*Bombyx mori*	Enter the pupal stage earlier	Trehalose, glycogen, lipids	Physiological regulation	Satake et al., 2000; Chen and Gu, 2006
12	*Manduca sexta*	-	Glycogen	Physiological regulation	Meyer-Fernandes et al., 2000
13	*Rhodnius prolixus*	-	Proteins	Physiological regulation	Paim et al., 2016


Insufficient food also limits insect reproduction (Zhang H. H. et al., 2015; Ojima et al., 2018). The most typical survival mechanism is to reduce reproductive investment under starvation conditions to increase somatic cell maintenance (Elkin and Reid, 2005; García-Roger et al., 2006; Billings et al., 2018). Studies of *Bactrocera minax* (Diptera: Tephritidae) indicated that hunger was not conducive to ovary development and led to a significant reduction in the number of matings and egg production and a prolonged spawning period (Huang, 2015). Similarly, under starvation conditions, *Arma chinensis* (Hemiptera: Pentatomidae) reduced the number of eggs laid and also exhibited a decline in the hatching rate of eggs (Zhang et al., 2017). Compared with bees kept under normal environmental conditions, the ovaries of hungry *Apis mellifera* (Hymenoptera: Apidae) worker bees showed shrinkage and decrease in egg plaques (Wang et al., 2016a,b). These behavioral adjustments are aimed at limiting population size to ensure availability of sufficient food for the existing population. However, there are exceptions. In the case of *Eriosoma lanigerum* (Hemiptera: Aphididae), the early stages of starvation produced a large number of breeding individuals followed by a sharp decline with progressive duration of starvation. In this way, the production of future generations was prioritized and then their own life processes were maintained (Chen, 2013).

In nature, most insects enter diapause during winter food shortages. However, a small number of insects migrate to places more suitable for growth and reproduction. After the low temperatures and food shortage conditions are over, they return to their original habitats to continue normal survival and growth (Feng et al., 2014). For example, in the fall in China, *Mythimna separata* (Lepidoptera: Noctuidae) moves from high to low altitudes and latitudes for the winter (Li, 1993). Incompletely metamorphosed insects that do not experience the pupal stage seek food by moving. It must be noted that hunger stress can also affect the ability of insects to take off and fly. Hunger before flight can have a negative impact on flight endurance (Kehl and Fischer, 2012), for instance, the speed and angular velocity of beetles decrease with increasing hunger (Nguyen, 2008). Despite these challenges, migratory movement is still one of the most important ways in which insects respond to hunger stress.

## Physiological Regulation of Insect Energy Under Starvation Stress

### Carbohydrates

Carbohydrates are a key source of energy for insects. Carbohydrates, mainly in the form of glycogen, trehalose and glucose, play an important role in energy metabolism and metabolite synthesis (Gaxiola et al., 2005; Kehl and Fischer, 2012). They not only enhance insect hunger resistance, but also play a vital role in other physiological adaptations (Tang et al., 2012b; Chng et al., 2017). Studies have found that starved larvae of *A. mellifera* can still maintain a stable blood sugar trehalose concentration (Wang et al., 2016a).

When the blood sugar content of insects is low, glycogen can be broken down, especially during starvation (Marron et al., 2003; Arrese and Soulages, 2010; Parkash et al., 2012; Tang et al., 2012a; Rovenko et al., 2015). Glucose is an equally important carbohydrate and all sugars are eventually converted to glucose to provide ATP, an energy source and metabolic intermediate in living cells (Jensen et al., 2015). Trehalose, an important component of insect blood is also known as the “sugar of life” (Shukla et al., 2015). In harsh environments, such as severe cold, high temperature and drought, trehalose accumulates and is used to maintain normal life processes and enhanced survival (Yu et al., 2008). Glycogen, glucose and trehalose can be converted into other forms for energy storage and release in insects (Tang et al., 2010). When insects are starved, trehalose is used first as a source of energy (Tang et al., 2014), leading to a rapid decrease in glucose and trehalose in their hemolymph (Satake et al., 2000). Under starvation stress, the trehalose content of *Trogoderma granarium* larvae was significantly reduced (Mohammadzadeh and Izadi, 2018). In starved *P. hilaris* larvae, glucose levels significantly decreased and the level of trehalose showed an initial decrease, followed by an increase (Munyiri and Ishikawa, 2005). The hunger-tolerance mechanisms of insects differ. For example, the hemolymph glucose concentration of *A. mellifera* was significantly reduced under starvation stress (Buckemüller et al., 2017) whereas the phosphorylase activity of *Bombyx mori* and *Manduca sexta* larvae increased and the glycogen content gradually decreased (Meyer-Fernandes et al., 2000; Satake et al., 2000). When the intensity of starvation stress increases, glycogen is converted to trehalose and released into the blood to maintain energy metabolism (Bede et al., 2007). Therefore, as the intensity of starvation increases, the levels of trehalose and glycogen in insects decrease, but trehalose remains at a relatively low and stable concentration (Shi et al., 2017). After relief from hunger stress, the hemolymph glucose levels of insects increase (Sánchez-Paz et al., 2007) and are gradually converted into trehalose and glycogen for storage.

The most important substances that mediate the decomposition of carbohydrates are trehalose synthase (TPS), trehalase (TREH), glycogen synthase (GS), and insulin-like pathway-related genes (Tang et al., 2012b). In *H. axyridis* exposed to starvation stress, the trehalose level and trehalase activity were significantly lower 8 h after starvation treatment, but the relative expression of *TRE1-1* increased. After 8–24 h of starvation, trehalose was maintained at a high level, but glycogen levels decreased. These results indicate that trehalose plays a key role in the starvation process through molecular and biochemical regulation of trehalose and glycogen metabolism (Tang et al., 2014; Shi et al., 2017). When human blood sugar levels are very low, the body increases the blood sugar concentration by regulating the insulin signaling pathway. For insects, insulin-like signaling pathways *in vivo* play a similar role in controlling the balance of blood glucose concentrations (Kuhn et al., 2015; Zhai et al., 2015). Similarly, in *D. melanogaster* and *B. mori*, a decrease in insulin-like signaling levels regulate the expression of related antimicrobial peptide genes (Becker et al., 2010; Yang et al., 2016; Lebreton et al., 2017). These genes participate in immune regulation and repair of damage and thereby enhance the resistance of insects to starvation (Riddell and Mallon, 2006).

### Lipids

Fat bodies are the main units of lipid storage in insects and their storage function is key for normal life processes (Ballard et al., 2008; Kehl and Fischer, 2012; Park et al., 2013). To combat hunger, stored lipid resources are often used through reduced glucose oxidation and increased fatty acid mobilization and lipid oxidation (Gergs and Jager, 2014; McCue et al., 2015; Wang et al., 2016a). Lipid metabolism in insects is mainly regulated by Adipokinetic hormone (AKH), the fat stimulating hormone, which is secreted under low nutrient conditions, thereby leading to lipolysis, glycogenolysis, and sugar and lipid nutrients moving from the fat body into the hemolymph (Kim and Rulifson, 2004; Lee and Park, 2004). AKH, a key regulator of energy—also mobilizes sugar and lipids from insect fat bodies during high-energy activities such as flight and exercise and contributes to the balance of hemolymph sugars, lipids, and carbohydrates (Staubli et al., 2002; Hou et al., 2017). Intensive research has found that the ability of insects to survive starvation is largely dependent on the ratio of triglycerides to lipids in the body (Renault et al., 2002; Ballard et al., 2008; Laparie et al., 2012). When insects fly under starvation conditions, lipids are the main source of energy for the flight muscles (Ryan and van der Horst, 2000). Lipids are, therefore, closely linked to insect movement during starvation. Of course, the use of lipids under starvation stress varies among different insects. Under laboratory starvation conditions, the concentration of lipids in the hemolymph of *B. mori* increased (Satake et al., 2000) whereas in *Pachnoda sinuata* (Coleoptera: Scarabaeidae), the lipids in the flight muscles and fat bodies significantly decreased (Auerswald and Gäde, 2000). The beetle *Merizodus soledadinus* (Coleoptera: Carabidae) significantly increased the hydrolysis of triglycerides during food deprivation which returned to normal levels after feeding (Laparie et al., 2012). This suggests that triglycerides are immediately mobilized to enhance hunger resistance in the event of food shortage.

It is worth noting that moderate hunger leads to the accumulation of fat, which is dependent on the developmental stage of the insect and the state of feeding (Lorenz, 2001). Increased fat mass in insects can increase resistance to hunger, indicating a significant correlation between lipid levels and hunger resistance (Parkash et al., 2014). Pure lipid-based triglycerides are used during starvation (Sinclair et al., 2011) and proteins that are starved by lipid synthesis or degradation are affected by the opposite form. For example, a fatty acid synthase and a glycocholine transfer protein were down-regulated four fold after 4 h of starvation, while triacylglycerol lipase levels increased 10 fold (Muhlia-Almazán et al., 2005).

### Proteins

A modest decrease in protein content during insect starvation indicates that insects can cope with hunger by using different endogenous reserves (Gäde and Auerswald, 2002; Helland et al., 2003). After starvation, the concentrations of alanine in the flight muscles, lipid bodies, and hemolymph of insects rapidly decline whereas those of proline remain high (Kehl and Fischer, 2012). At the same time, the heat shock proteins 70 (HSP70s) are important stress protectants in *H. axyridis* and *Rhodnius prolixus* (Hemiptera, Reduviidae) and play a role in adaptation to food deprivation (Shen et al., 2015; Paim et al., 2016). In addition, studies have shown that the positive effects of adult amino acids are limited to females, presumably because their high protein demand significantly changes the catabolic rate (Mevi-Schütz and Erhardt, 2005; Bauerfeind and Fischer, 2009). There is ample evidence that amino acids extracted from protein breakdown are degraded during starvation (Haubert et al., 2005).

### Nerve Signals and Hormones

When insects are stressed by hunger, they initiate a series of anti-starvation mechanisms. These mechanisms include regulating the expression of related genes, synthesizing anti-stress substances and regulating the catabolism of energy substances in the body to maintain normal growth and development (Buckemüller et al., 2017). A study in *D. melanogaster* found that dG9a (histone methyltransferase) is a key factor in tolerance to hunger stress and is also a key regulator of behavioral strategies under starvation conditions (An P.N.T. et al., 2017; Shimaji et al., 2017). Hunger is a powerful driver of food intake and some neurons, neuropeptides and neurohormones play key roles in behavioral and physiological regulation (Perić-Mataruga, 2006; Jourjine et al., 2016; Mena et al., 2016). At the same time, starvation increases the expression level of SLC5A11 neurons and enhances their excitability by inhibiting the dKCNQ channel, thereby conferring hunger status and promoting feeding and starvation-driven behaviors (Park et al., 2016).

Hunger itself affects stress in insects and the expression of immunity-related genes. Many immunity-related genes, such as interleukin 1-β, are significantly down-regulated during starvation (Riddell and Mallon, 2006; Buckemüller et al., 2017) leading to lower metabolism followed by a concomitant decline in immunity (Guo et al., 2014). Starvation stress can also significantly up-regulate the expression of two octopamine (OA) receptor genes (Li, 2017). OA has significant biological effects on the growth and behavior of various arthropods and poor living conditions pose differential consequences on the distribution and content of OA. Hunger not only has an effect on the transcription of the brain and surrounding tissues in *Drosophila* (Bos et al., 2016; Singh et al., 2018), but also induces autophagy in *Drosophila* larvae during metamorphosis by inhibiting the PI3KI/Akt-Tor pathway (Riddiford et al., 2000; Wang et al., 2012). Similarly, starvation can also induce autophagy in *Spodoptera frugiperda* (Lepidoptera: Noctuidae) Sf9 cells (Xie K. et al., 2017). Increased levels of autophagy are usually induced by signals such as starvation, while excessive levels of autophagy can cause autophagic programmed cell death. During starvation, the level of autophagy helps to reduce the level of apoptosis in fat cells, thus playing a protective role in the survival of adipocytes (Otomo et al., 2013; Li et al., 2015).

Dopaminergic signaling pathways and juvenile hormones (JH) are also important stress-resistance substances in insects and play a role in adaptation to hunger stress (Neckameyer and Weinstein, 2005; Lee and Horodyski, 2006). While the former directly affects the metamorphosis and development of insects (Yang, 2014), the latter confers damage protecting (Shi et al., 2016). After starvation treatment, the growth and development of *Plutella xylostella* (Lepidoptera: Plutellidae) were found to be delayed because the expression of the juvenile hormone acid methyl transferase (JHAMT) candidate gene *Px009591* increased and the expression of the juvenile hormone esterase (JHE) gene *Px004817* and JHE activity decreased. These changes led to an increase in JH levels in insects, which in turn delayed growth and development (Duan, 2016). Similarly, the rate of biosynthesis of *M. sexta* gradually increased after starvation (Lee and Horodyski, 2006). Telang et al. (2010) also found that JHE mRNA levels in the 4^th^ instar larvae of *Aedes aegypti* (Diptera: Culicidae) were close to zero after 36 h of starvation. In addition, when the expression of the hsp18.3 gene of *Tribolium castaneum* (Coleoptera: Tenebrionidae) was silenced by RNAi technology, the resistance of the starved group was significantly lower than that of the control group (Xie J. et al., 2017). Similarly, a study of three *HSP70* genes in *H. axyridis* showed that their relative expression not only increased with increasing temperature, but also at the peak of starvation at 8 h (Shen et al., 2015). Thus, juvenile hormones and heat shock proteins have a significant effect on insect emergency stress responses.

## Future Prospects

Nutrition plays an important role in the life history of insects, especially as reproduction is influenced by the quality and quantity of food (Fischer et al., 2004). While dietary restrictions can reduce reproductive yield, it can also extend an insect’s lifespan (Partridge et al., 2005; Carey et al., 2008). At the same time, insects exposed to starvation or low temperatures may enter a state of diapause. In previous studies, 46 diapause-related genes were found in *Aphidius gifuensis* (Hymenoptera: Aphididae) (Huang et al., 2015; An T. et al., 2017) and 443 in *Coccinella septempunctata* (Coleoptera: Coccinellidae) (Liu et al., 2014; Ren et al., 2015; Qi et al., 2016). These genes are associated with energy demand during diapause and inhibition of metabolism. During diapause and wintering in insects, nutrient levels, amino acid accumulation and transformation and regulatory mechanisms including insulin signaling pathways are similar to those under starvation stress (Hahn and Denlinger, 2007; Huang et al., 2015; Ren et al., 2016; Sinclair and Marshall, 2018). Moderate hunger can also have a positive impact on insects and on pest control. Thus, a systematic examination of behavioral and physiological strategies under starvation stress can provide significant theoretical basis for the development of natural breeding in insects and storage of natural enemy insects for their use in pest control.

Nutritionally challenging situations in environments have evolved insects with diverse adaptive responses to withstand periods of food shortage. In this review we have attempted to showcase the starvation stress physiology of insects with respect to behavioral attributes and physiological regulation. Given their broad range of ecological habitats, insects are suitable and convenient models to evaluate the strategies they employ to cope with starvation. Moreover, starvation stress research in insects offers interesting cues from ecological and evolutionary perspectives that not only govern reproduction, survivorship and cross tolerance to other environmental stressors but also dictate phenotypic plasticity in adjusting development and life histories around nutritionally optimal environmental conditions. Such studies are critical to our understanding of starvation-induced physiological responses that precede death and in extrapolating them to vertebrates.

## Author Contributions

B-PZ, Z-JX, KL, and D-WZ conceived and manuscript structure design. B-PZ, Y-LT, and D-WZ wrote the manuscript.

## Conflict of Interest Statement

The authors declare that the research was conducted in the absence of any commercial or financial relationships that could be construed as a potential conflict of interest.
